# Quantification of landfill gas emissions and energy production potential in Tirupati Municipal solid waste disposal site by LandGEM mathematical model

**DOI:** 10.1016/j.mex.2022.101869

**Published:** 2022-09-20

**Authors:** C. Ramprasad, Hari Charan Teja, Vunnam Gowtham, Varadam Vikas

**Affiliations:** a School of Civil Engineering, SASTRA Deemed to be University, Thanjavur 613 401, Tamil Nadu, India; b Centre for Advaced Research in Environment (CARE)

**Keywords:** Energy production, Landfill, LandGEM model, Municipal solid waste: Methane emissions, Tripathi, Waste management

## Abstract

The present key challenges the world is currently facing are the environmental pollution, climate change and energy crisis. The anthropogenic emissions of carbon dioxide due to burning of fossil fuels for energy production and other greenhouse gas emissions are considered unsustainable, and whole world is having a paradigm shift towards the renewable energy. The one of the major contributor of the greenhouse gases like methane, carbon dioxide are the municipal solid waste landfill sites. The landfill sites contains nearly 50–60% of organic contents, and they undergo anaerobic decomposition with a help microbes in the waste dumps contribute to the higher percent of methane emissions. There is now enhanced public awareness on sustainable products, and commodities usage in their daily needs, hence the global warming can be slowed down and devise an environmentally sound sustainable society. The present study aimed to provide a methodology to quantify the amount of methane and carbon-di-oxide emitted from the Tirupati municipal solid waste dumpsite using LandGEM3.02 model and empirical equation to estimate the renewable energy potential. The method provided was simple and more accurate having higher efficiency in predicting the landfill emissions and subsequently the energy potential. The study shows that the energy emission potential are maximum to the waste with a higher fraction of biodegradable organic content. Therefore, the method can be implemented in all the landfills by the policy makers to predict the methane emissions and control the greenhouse gas emissions by sequestering the methane and carbon dioxide optimally for energy production.

• Landfill gases are a primary constituent in greenhouse gases and has potential for energy production.
• The results from this study showed an abundant quantity of methane and carbon dioxide are emitted from tripathi landfill site.
• The study concludes that methane can be extracted and used as alternative source of sustainable energy.

## Graphical abstract

**Figure:**
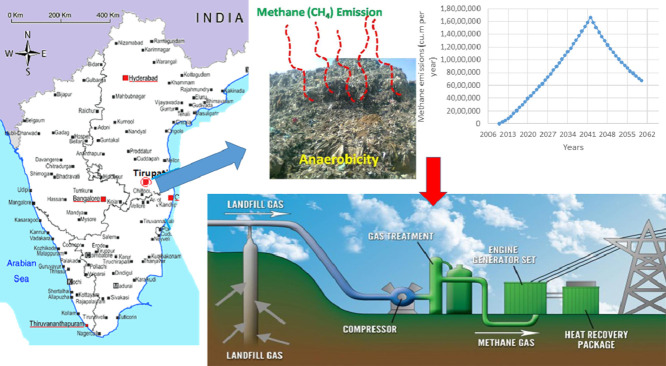

Specifications Table

**Table:** 

Subject area:	Environmental Science and Engineering
More specific subject area:	Landfill gas emissions
Method name:	Estimation of methane gas emissions from landfill site and quantify the energy potential using empirical equations
Name and reference of the original method:	Ghosh, P., Shah, G., Chandra, R., Sahota, S., Kumar, H., Vijay, V. K., & Thakur, I. S. (2019). Assessment of methane emissions and energy recovery potential from the municipal solid waste landfills of Delhi, India. *Bioresource technology*, *272*, 611–615.
Resource availability:	The data are available with this article.
Submission Type:	Direct Submission

## Method details

The increasing population and the growth in socio-economic status of the people resulted in a huge production in Municipal Solid Waste (MSW). India is one of the fastest developing country in the world with an expected population of 1400 million during the year 2026 [32]. As per the Central Pollution Control Board [10], the urban India generated 62 MT of municipal solid waste in the year 2015 and projected to be reach 165 MT at the end of 2030 [3,32]. The CPCB report showed that the municipal solid waste generation rate lies between 0.2–0.3 kg per capita per day in small towns/cities with populations less than 0.2 million. It is usually 0.4–0.6 kg per capita per day in cities with population ranges of 200,000 - 1 million [10], [21], [32]].

Tirupati is one of the fast growing, a major pilgrimage and a cultural heritage city of the Indian state, Andhra Pradesh with the rapid development in industrial sectors, medicinal fields, and other services have led to rapid population growth. The city had a population of 2, 96, 156 with a surface area of 16.59 sq. km and a yearly average precipitation of 905 mm [8]. It was projected that the city will have a population of 6,62,975 about a growth rate 32%, will further add to the amount of solid waste production. Recently, the Government of India (GoI) has initiated the policy named as “Swachh Bharat Mission” with a vision to clean India and special emphasis was provided for MSW management. Nevertheless, there are many sound technologies and expert engineers to handle the MSW; the socio-economic problems prevent the comprehensive waste management plan. Many researchers show that landfill are most economically, technically feasible and socially acceptable one, has a high paid attention in the developing countries for the management of solid waste [13,14,22,28]. The landfill operation, climatic conditions and characteristics of solid waste dumped leads to a major problem of concerns within the landfills like the emissions of landfill gases and high amount leachate generation [14,26,39].

Landfill gases (LFG) are produced due to the decomposition and bio-chemical reactions occurring to the waste dumped in the landfills. The primary landfill gases that are emitted are 40–50% of carbon dioxide and 50–60% of methane [19,24,33]. The wastes dumped in the landfills undergo anaerobic decomposition due to the microbes present and start emitting landfill gases [14,25]. The landfill gases such as methane and carbon dioxide are the primary constituents responsible for greenhouse effect. The global warming potential of the landfill gases are much higher especially, methane has a 21% higher potential than carbon dioxide to cause the global warming effect [1,40]. The existing dumpsites in India are not properly managed, sometimes the waste are not compacted as well as daily cover of 10–15 cm soil were not provided. The above condition paves way for the odour problem, continuous release gases pollutants into atmosphere and fire outbreaks [28]. There are several studies highlighting the problem of landfill gases and quantified the levels of pollutants from the municipal solid waste dumpsites using diverse modelling techniques [13]. The landfill gases emissions depend on many factors such as local climatic conditions, waste proportions or characteristics, waste quantity, disposal pattern, and other protective systems. The modelling approach should consider all the above factors and should precisely predict the emissions rate as it is very significant for designing and operating the MSW landfill sites [38], especially for Tirupati landfill site. The prediction of total methane emissions from the Tirupati landfill site could help in determining the greenhouse emissions from India and the potential for sustainable energy source.

The methane emissions from the landfills can be estimated from various mathematical modelling methods as well as field measurements [11], [13], [23]. There are models that predict the emission rate more precisely and substantiate the field results [7]. The most common and widely used models are IPCC default method, Triangular method, SWEET model, and First order decay model. There are many limitations with the above methods and huge uncertainties in predicting the landfill gases from the actual disposal. Additionally, there are lack of data with respect to MSW generation, disposal, and country specific hydro-climatic factors [20,32]. The Landfill Gas Emission Model (LandGEM) was studied by few researchers and reported to be fairly better model for the prediction and estimation of LFGs [2,13,34]. The study conducted by Sil et al., [34] using the LandGEM showed that, the methane emissions for the two cities in Mumbai was 5524 and 5630×10^6 cu. m/ year. In an another study, the methane emissions for the year's 2018, 2023, 2028, and 2033 were 205, 410 549, and 671 cu. m per year respectively [30]. Many other researchers also studied on methane emissions estimation using LandGEM from Indian cities, but no research have been carried out in Tirupati. Therefore, the present study aims to quantify the amount of landfill gases such as methane, carbon dioxide, and carbon monoxide emissions as a potential energy source as well as carbon reduction from Tirupati municipal solid waste landfill site using the LandGEM 3.02 model.

## Materials and methods details

### Study area

**Fig. 1:**
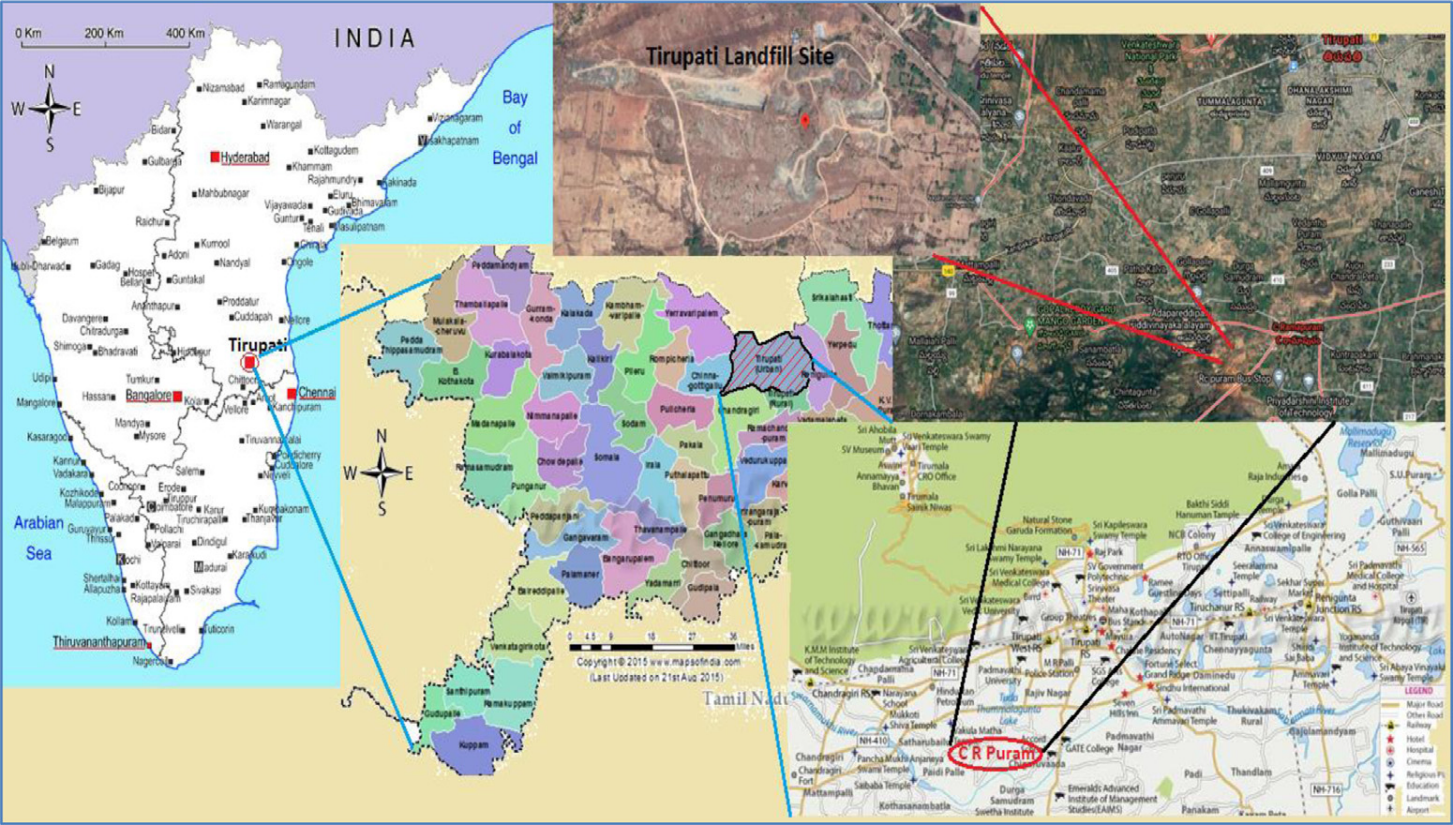
Photograph of the Tirupati municipal solid waste dumpsite.

The present study was conducted on a municipal solid waste dumpsite located in Tirupati, Andhra Pradesh, India. The solid waste from six clusters - II towns such as Tirupati, Srikalahasti, Puttur, Nagari town, Chittoor and Venkatgiri were collected and disposed in the trench landfill. The site specific parameters such as groundwater level, annual precipitation rate, population data, surface area, average annual temperature, solid waste generation rate per capita and quantification of waste are collected and tabulated in Table 1. The solid waste generated from all the six clusters were collected and transferred to CR Puram, approximately 12 km away from the centre of the city. The GPS coordinates for the Tirupati dumpsite i.e., CR Puram are 13°06′22.9″N 79°10′00.3″E and the view of the dumpsite are shown in Fig. 1. The waste dumping of 160 tonnes per day (TPD) were done in the open non-engineered landfill trenches, and organic biodegradable fractions were processed in a vermin-composting plant. Therefore, prone to ground water and soil pollution because of leachate generation is higher. Open dumping of waste facilitates vector nuisance, odour problem, becoming breeding grounds for mosquitoes leading to several health issues.

**Table 1:** Site characteristics of Tirupati municipal solid waste dumpsite.

Sl. No	Characteristics / Factor	Values
1.	Surface Area	16.59 sq. kms
2.	Population	3,82,934 (Census, 2011)
3.	Annual precipitation	905 mm
4.	Average annual temperature	28.4 °C
5.	Solid waste generation rate	0.46 kgs per capita per day
6.	Quantity of waste	195 TPD
7.	Groundwater level	35–50 m

### Data collection

The CR Puram landfill started to receive the municipal solid wastes collected from the municipalities from the year 2010 by various transport mechanics such as pushcarts, tractor trolleys, and auto-tippers. The data pertaining to the landfill site was collected from the Tirupati Municipal Corporation office and are presented in the Tables 1 and 2. The data's show that the average annual growth rate of solid waste was 1.3% and projected to reach 250 tonnes per day by 2025. The amount of waste collected and disposed in the dumpsite are obtained for every year (2010 – 2019) from the municipal office and were projected for the future by considering the selective growth factor and the factors influencing the population growth rate using the arithmetic progression method. The landfill gaseous emissions were determined by the LandGEM model using the yearly mass of waste deposited and the corresponding methane emission potential. The solid wastes are dumped adopting the trench technology, the landfills are excavated for a depth of 3 m and were compacted. The available length and width in the CR Puram landfill is 400 m and 300 m respectively, and the buried waste are to be daily covered with a normal soil to a depth of 15–30 cm according to the CPHEEO standards. The natural of soil type in the landfill site is sandy-loam soil.

**Table 2:** LandGEM model parameters and specification used in the present study.

Notation	Parameter Name	Value	Unit	Reference
$Q_{CH_{4}}$	Quantity of annual methane generation from landfill	–	cu. m of methane per year	[13,36]
k	Rate constant for methane production	0.05	per year	
Lo	Methane production potential	110	cu. m per Mg	
Mi	the mass of waste accepted in the i^th^ year	–	Mg	
	Methane content	50	% of volume rate	
NMOC	Concentration of non-methane organic compounds	4000	ppmv	
	Beginning year of landfill disposal	2010	Year	Tirupati municipal corporation
	Landfill closure year	2041	Year	
	Does the year of landfill closure require a computing model?	NO	–	
	Landfill capacity	NA	Mg	
	Landfill design life	30	years	

### Description of LandGEM model

The United States Environmental Protection Agency (USEPA) has developed a first order decomposition rate model named as LandGEM to quantify the amount of landfill gas emitted from the municipal solid waste dumpsite. The Landfill Gaseous Emission Model (LandGEM 3.02) is relatively simple and uses an empirical equation to estimate the landfill gas (LFG) emissions. The Eq. (1), adopts two important parameters to predict the LFG, they are methane production first order rate constant (k in per year) and methane production potential (Lo in cu. m per Mg). The amount of methane generation in a year is given by the following equation and the model parameters incorporated in the present study are tabulated in the following Table 2. The LandGEM model predicts the landfill gases such as methane, carbon dioxide and carbon monoxide for the design life period and its potential source of energy.

(1) $$Q_{CH_{4}}=\;\sum_{i=1}^{n}\sum_{j=0.1}^{1}kL_{o}\left(\frac{M_{i}}{10}\right)e^{-kt_{ij}}$$

Where, i is the one-year time increment and j is 0.1year time increment n defines as (year of the calculation) - (initial year of waste acceptance). t_ij_ is the age of the j^th^ sector of waste mass, M_i_ accepted in the i^th^ year

The sequential procedure that was adopted in the present study to collect the data's pertaining to dumpsite and modelling the obtained data using the landGEM model are discussed as follows. Initially, the population data, quantity of solid waste generated, percentage of waste collected from the city, location of dumpsite, per capita waste generation rate, solid waste composition, landfill inception year, and landfill capacity were collected as a document from the Tirupati Municipal corporation office. Secondly, the team members visited the CR puram dumpsite, located the GPS coordinates, assessed the site conditions, and photograph's of the site. Subsequently, the population was projected till the design life of the landfill using the arithmetic progression method considering the latest growth rate and the waste generated also calculated using the per capita waste generation rate. At the next, the data's like quantity of waste deposited into the landfills, inception year and closure year are entered into the LandGEM excel software. The software's calculates the methane, carbon dioxide and total landfill gases using the default values like k, Lo values and the methane content as shown in Table 2. Finally, the estimated methane concentrations from the landGEM model was used to calculate the amount of electrical energy produced as per the empirical Eq. (2).

### Energy generation potential from methane

The potential energy generation of the emitted methane from the year 2010 to 2042 was estimated using an empirical Eq. (2). The most vital parameter that governs the energy generation potential from landfill is the recovery efficiency. Generally, in a landfill site the generation of energy from all the produced gases is not practical, there will be some losses during the extraction process. According to [31], the collection efficiency was in the range of 70–75% and the lower heating value (LHV) of methane is 15–35 MJ per cu. m. In the present study, the collection efficiency of 70%, a LHV of 18 MJ per cu. m was considered, and the Internal combustion engine (ICM) is considered for its cost effectiveness and higher conversion efficiency of 40%. The energy potential for the generated methane is calculated by the following Eq. (2),

(2) $$E_{p}\left(kWh\;per\;year\right)=\;\frac{LHV*\;Q_{rg}*\;E_{e}*\;E_{r}}{\gamma_{i}}$$

Where,

E_p_ is the energy potential that can be obtained by the methane gas in kWh per year

Q_rg_ is the quantity of recoverable methane gas emitted from landfill site in cu. m per year

LHV is the low heating value of the methane gas in MJ per cu. m

E_e_ is the electrical combustion efficiency of the engine element (ICM) in percentage

E_r_ is the efficiency of the methane recovery in percentage

γ_i_ is the conversion factor from MJ into kWh (1 MJ is equal to 0.278 kWh)

In the article [9], the energy potential was calculated using an empirical equation as shown in Eq. (3). The equation used in the above article nudged to a question on the use of two empirical coefficients. The empirical coefficients used in the above article were 0.9 and 3.6, which can be improved into a single numerical. The coefficient 0.9 is a constant value provided in the article [29] and [5] and coefficient 3.6 is the conversion factor from MJ to kWh.

(3) $$E_{p}=\;\frac{0.9\times \;Q_{\text{methane}}\times \;\text{LH}V_{\text{methane}}\times \eta \times \lambda }{3.6}$$

Therefore, in the present study the empirical coefficients were combined into a single numerical as 0.278. The improvement in the methodology part was in the empirical coefficients by merging them considering the values as 0.997 and 3.6 respectively. In the present study, the energy potential equation was simplified into a notation form and empirical constants were implicitly included into the notations (γ_i_). In the present study, the value 0.9 is modified or improved to 0.997, because previous authors have considered a landfill with a cover. Hence, 10% of oxidation due to cover was considered in the study, whereas in the present study area (Tirupathi), there is no practise of landfill cover, so no or less oxidation will occur for the solid waste dumped. Hence, a mere percentage of 0.3 was considered as oxidation due to the atmosphere and climatic conditions.

## Results and discussion

### Solid waste quantification

The solid waste generated in the Tirupati was characterized for various physico-chemical parameters by the Tirupati Municipal Corporation (TMC). The mixed solid waste obtained from household, market, temples and commercial places showed an average bulk density of 359.33 kg per cu. m, moisture content of 27.6%, pH of the mixed waste was slightly alkaline in nature and the value is 7.95, carbon to nitrogen ratio was 30.03 and calorific value was 1324.67 kcal per kg. The municipal solid waste collected in the Tirupati contains the following components, 53.3% of biodegradable fractions like food waste, vegetable waste, etc., followed by 26% of recyclables like papers, cardboards, plastics, etc., 13.1% of inert, dry leaves of 2.7% and coconut shells from temple contribute to 4.9%. The composition of the waste is in good agreement with [37] as the author said that major portion of the waste generated is organics.

The methane potential of the waste depends highly on the anaerobic microbe's presence and biodegradable waste portion. Additionally, the factor such as climatological conditions, volatile fatty acids, and segregation accuracy also contributes to a slighter fraction for the methane production [13,27]. The quantities of waste collected during each year from various zones were tabulated in Table 3. The waste generated in the TMC are discarded in the landfills, during the year 2020 the quantity of waste generated was 80,511 Mg per year and projected for the next 20 years (upto 2041) and found to be 1,81,565 Mg per year. The cumulative of waste in-place in the dumpsite were found to be 5, 60,432 Mg per year during the 2020, and were found to be 31, 26,706 Mg per year during 2041. After the waste acceptance was stopped the landfill contains the solid waste of 33, 08, 272 Mg per year and remained constant for several years. These data show that the rapid increase in municipal solid waste productions in the study area are due to the population growth and increasing industrial and commercial establishments.

**Table 3:** Quantity of waste generated from Tirupati Municipal Corporation.

Year	Amount of waste accepted(Mg per year)	Year	Amount of waste accepted(Mg per year)	Year	Amount of waste accepted(Mg per year)
2010	30,768	2021	82,970	2032	128,838
2011	32,375	2022	84,783	2033	132,656
2012	41,514	2023	87,438	2034	139,056
2013	46,620	2024	90,677	2035	145,071
2014	59,984	2025	98,781	2036	147,697
2015	64,994	2026	100,344	2037	159,299
2016	66,778	2027	105,293	2038	165,529
2017	69,507	2028	112,716	2039	169,395
2018	71,933	2029	117,252	2040	173,944
2019	75,960	2030	120,166	2041	181,565
2020	80,511	2031	123,858	2042	0

### Landfill gas emissions

**Fig. 2:**
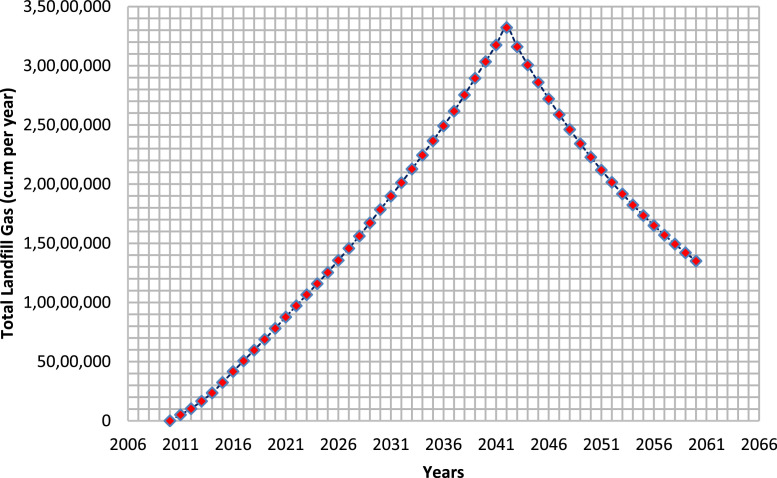
Total Landfill Gas emissions in cu. m per year from Tirupati dumpsite over 2010–2060.

The landfill gases (LFG) such as methane, carbon dioxide, non-methanogenic organic compounds and carbon monoxide are considered to have high potential for global warming as they are categorized as greenhouse gases. The global warming potential of methane over the 20 years’ span was found to be 84–86 [15]. The solid waste that are disposed in the study area are predominantly organic and due to anaerobic microbe's presence the decomposition occurs and resulted in the emission of methane and carbon dioxide. The landfill gas emission highly depends on the half-life of the organic matter like food waste. During the year 2010, the total quantity of waste disposed was 30,768 Mg per year (Table 3), and was increased to 80,511 Mg per year during 2020. It was projected that, the quantity of waste generated in the city will reach 1,73,944 Mg per year by the year 2040. The methane emission that was estimated by the LandGEM model varies according to the landfill management and waste composition [13]. According to the Fig. 2, the total gas emission from the disposed waste started to increase gradually over time and reached a peak at 2042. The total production of landfill gases in the year 2011 was 5, 11, 474 cu. m per year and reached to 78, 09, 589 cu. m per year by the end of 2020. Furthermore, it can be shown that the production of total landfill gases increased from 0% to 6.55% within 10 years. The total gas emissions dropped after 2042 due to landfill closure, the results were in good agreement with [13,30] and [2].

The calibration and validation of landGEM model was researched earlier by [41], [42], [43]. The researchers showed that the actual landfill emissions measured in the field from the Halton landfill, Ontario, Canada was slightly lesser than the LandGEM model predicted values. According to the study, the k value of 0.04 per year and Lo value 101 cu. m per ton gave a good correlation [42]. Therefore, present study also used the Lo value 110 cu.m per year and k value of 0.05 per year, to substantiate the previous studies.

**Fig. 3:**
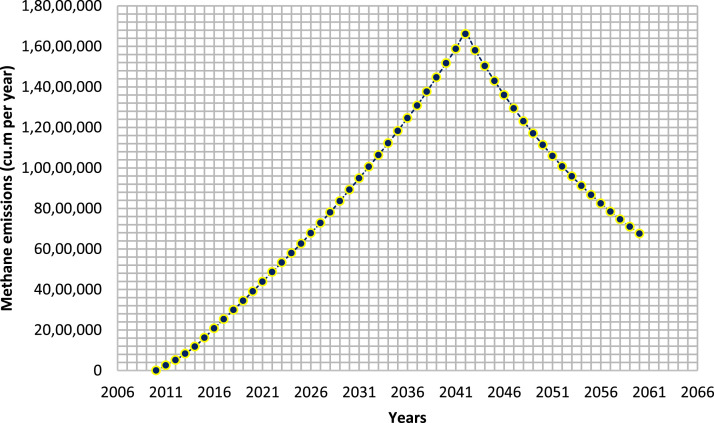
Methane emissions in cu. m per year from Tirupati dumpsite over 2010–2060.

At the Tirupati solid waste disposal site, the methane gas emission trend was depicted in Fig. 3 over the years from 2010 - 2060. Similar to total landfill gas emissions, the methane emissions showed an increasing trend till 2042 and then started to decrease. During the year 2011, the methane emissions was 2, 55, 737 cu. m per year and reached to 39, 04, 794 cu. m per year during 2020. The above obtained results are in accordance with the Kerman, Sanandaj and Yasuj landfill sites. Sadeghi et al. [30] showed that methane emissions during the first year of landfilling in Sanandaj city was 10 cu. m per hour and reached to 671 cu. m per hour after 20 years. Whereas, in the Yasuj city the methane emissions were slightly lesser with 5 cu. m per hour in the initial stages and 275 cu. m per hour after 20 years of landfilling [13]. The methane generation rate depends on the value of ‘k’ the emission rate constant as well as the amount of organic waste dumped. As the k value increases the production of methane also increases and the k depends on following factors such as pH of the waste dumped in landfill, moisture content of the waste, carbon to nitrogen ratio to decompose the waste into carbon dioxide and methane, and the temperature of the waste [34]. The methane also has a high calorific value of 20.5 MJ per cu. m and can be utilized as an alternative source of renewable energy [4].

However, the model predicted the landfill gases emissions rate, there are few limitations with the model. The assumption of empirical coefficients “Lo” and “k” as they are fixed over the entire life of a landfill site. It's a challenging assumption, as the waste composition, moisture content, nutrients, or climatic conditions vary over time, hence a fixed value is not an optional. In order to overcome the challenge, our future study focuses on using a “k” value obtained from a lab scale study and “Lo” value using an equation considering the biochemical methane potential values.

**Fig. 4:**
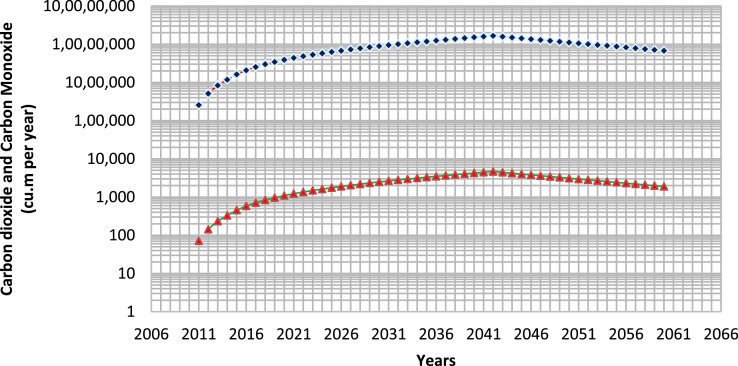
Carbon dioxide (Blue Diamond shape) and Carbon monoxide (Red Triangular shape) emissions in cu. m per year from Tirupati dumpsite over 2010–2060.

The carbon dioxide (CO_2_) one of the most vital greenhouse gases that is emitted from the landfill site due to the biodegradation of the solid wastes. The following Eq. (4), shows the stoichiometric emissions of CO_2_ from the organic wastes. The Fig. 4 shows the amount of carbon dioxide and carbon monoxide (CO) emissions over the years from the Tirupati dumpsite. The peak amount of carbon dioxide and carbon monoxide emissions were found to be during the year 2042, with 1.661E+07 cu. m per year and 4.651E+03 cu. m per year respectively. Similar to the methane, the CO_2_ and CO emissions also decreased after the closure year, it implies that emissions depend on the waste quantity, and biodegradation rate.

(4) $$C_{a}H_{b}O_{c}N_{d}+nH_{2}O\to xCH_{4}↑+y\;CO_{2}↑+wNH_{3}↑+zC_{5}H_{7}O_{2}N+energy$$

It is a well-known fact that emission of carbon dioxide into the atmosphere anthropogenically causes global warming and ozone layer depletion [6,16]. In the Tirupati landfill site, if the carbon dioxide emissions are sequestered by trapping the gas emissions and utilizing for energy generation leads to a clean development mechanism scenario. These approach was already being researched by many of the researchers all over the world [2,12,35].

### Energy recovery potential from MSW of the Tirupati landfills

**Fig. 5:**
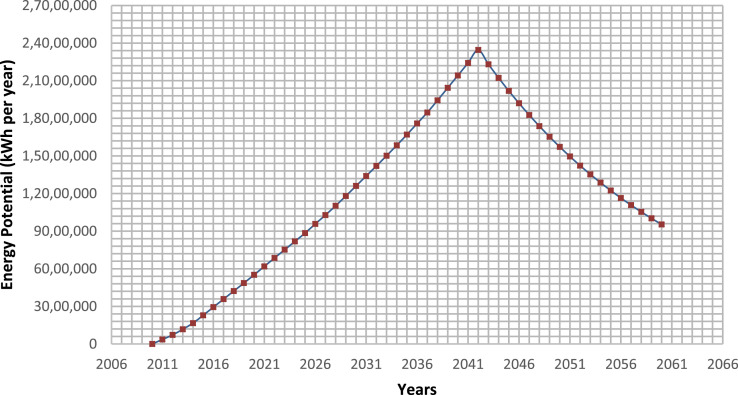
Energy potential in kWh per year from Tirupati dumpsite over 2010–2060.

According to United Nations mission on the sustainability development goals (SDG-17), the main objective was to use waste as a resource, develop a clean energy perspective to focus on sustainability. Therefore, the energy generation potential from the methane gas emitted from the Tirupati landfill site was estimated for a design life of more than 30 years (2010–2042), so as to get a perception into the use of municipal solid waste for energy recovery. It was predicted by the LandGEM model and Eq. (2), the methane emissions and energy potential showed a peak emission during the year 2042 and was found to be 1.66E7 cu. m per year and 2.09E8 MJ respectively. After the landfill closure, a decline in the energy potential was observed and was observed in the year 2050 (1.4E8 MJ). Furthermore, the electricity equivalent (kWh) was estimated for the Tirupati dumpsite based on the Eq. (2) and values provided in section 2.4. During the year 2020, the cumulative electricity equivalent (kWh) was calculated to be 5.51E6 and reached a peak (2.35E7) at 2042 (Fig. 5). The obtained results are in good agreement with the earlier studies [14,17,18,38]. According to a study conducted by Council of Energy, Environment and Water (CEEW), India between the period 23 April −23 May 2020 it was found that an average household consumes nearly 5.7 kWh per day. Assuming a town / village with 1500 equivalent houses, will roughly requires 3.12E6 kWh per year. The above calculation indicates that by harnessing the energy from the landfills, it not only reduces the emission of greenhouse gases into the atmosphere as well as acts like an alternative energy source for the households living close to the landfill site.

## Conclusion

In the present study, the landfill gas emissions for the Tirupati dumpsite were estimated using the LandGEM model. The landfill gases such as methane, carbon dioxide and carbon monoxide were projected from the inception year 2010 to 2060 more than the closure period. The landfill received the solid waste generated from the city and were directly dumped into the land without proper protections. The methane production from the dumpsite was projected to be 2.56E5 cu. m per year during the first year of waste acceptance. The peak emission of methane, 1.66 E7 cu. m per year was observed during the year of closure 2042. The other landfill gases such as carbon dioxide and carbon monoxide emissions were peak emitted during 2042 and the values were 1.66 E7 cu. m per year and 4.65E3 cu. m per year respectively. Additionally, the current research also concludes that Tirupati dumpsite has an energy potential of generating 44.62 kWh per minute if properly designed a gas collection system and maintained. Moreover, the present study also shows that the percentage of global greenhouse gases emissions from Tirupati dumpsite, which can be converted into a clean development mechanism by trapping the gases.

## Declaration of Competing Interest

The authors do hereby declare that, they have no conflict of interests.

## Data Availability

Data will be made available on request.
